# Inhibiting Interleukin 17 Can Ameliorate the Demyelination Caused by *A. cantonensis* via iNOS Inhibition

**DOI:** 10.1155/2017/3513651

**Published:** 2017-12-18

**Authors:** Feng Ying, Zheng Cunjing, Feng Feng, Wan Shuo, Zeng Xin, Xie Fukang, Wu Zhongdao

**Affiliations:** ^1^Medical School of South China University of Technology, Guangdong 510006, China; ^2^Histology and Embryology Department of Zhongshan School of Medicine, Sun Yat-sen University, Guangzhou 510080, China; ^3^The Department of Pharmacology and Toxicology, School of Pharmaceutical Sciences, Sun Yat-sen University, Guangzhou 510080, China; ^4^Parasitology Department of Zhongshan School of Medicine, Sun Yat-sen University, Guangzhou 510080, China

## Abstract

*Angiostrongylus cantonensis* (*A. cantonensis*) is an important food-borne parasitic disease. Previous study showed that *A. cantonensis* infection can cause demyelination in the central nerve system, but the mechanism of action has not been understood. To explore the mechanism and to look for effective therapeutic methods, interleukin 17A (IL-17A) and iNOS expressions were detected during *A. cantonensis* infection. In addition, IL-17A-neutralizing antibody was applied to treat *A*. *cantonensis-*infected mice. In our results, we found that IL-17A and iNOS RNA expressions increased gradually in the process of *A*. *cantonensis* infection. When infected mice were treated with IL-17A-neutralizing antibody, the pathologic changes of demyelination alleviated obviously, followed with the elevation of myelin basic protein (MBP) in the brain. In addition, the iNOS expression of the brain in infected animals also showed a decrease in astrocytes. Our study provided evidence that IL-17A may take part in the demyelination caused by *A*. *cantonensis* and inhibiting IL-17A expression can ameliorate the pathologic changes of demyelination. Moreover, the decreasing of iNOS expression may be the key reason for the effect of IL-17A inhibition on demyelination caused by *A*. *cantonensis.*

## 1. Introduction

*Angiostrongylus cantonensis* (*A. cantonensis*) is an important cause of food-borne diseases and eosinophilic encephalitis in humans [1]. *A. cantonensis* is a parasitic nematode from rats which invades the central nerve system (CNS) and causes eosinophilic encephalitis or meningoencephalitis [2]. During this process, neurons in CNS appear with obvious demyelination [3–5] (MBP is one component of myelin sheath) [6]. However, the reason for demyelination associated with *A. cantonensis* infection has not been fully known.

Cytokines of the interleukin 17 (IL-17) family are uniquely placed on the border between immune cells and tissue. As seen in psoriatic skin lesions or in joints of rheumatoid arthritis patients, high levels of IL-17 have been detected in CNS during inflammatory responses. Previous study showed that IL-17-induced Act1-mediated signaling cascades in CNS resident cells (astrocytes, oligodendrocytes, and neurons) might coordinately mediate CNS inflammation, demyelination, and neurodegeneration [7, 8]. But whether IL-17 is involved in the demyelination caused by *Angiostrongylus cantonensis* has never been studied.

Astrocytes probably represent the best-studied CNS resident cell type in the context of multiple sclerosis (MS) and EAE, which cause demyelination complications. Both human and mouse astrocytes (glial fibrillary acidic protein (GFAP) is the specific marker for astrocytes) express the IL-17RA, thereby allowing IL-17A ligation and consequently, the production of cytokines and chemokines, including IL-6, TNF*α*, and CCL2 [9–11]. Inducible nitric oxide (iNOS) is involved in various physiological regulations and plays important roles in some CNS disease, such as brain ischemia, brain infections, and neurodegenerative diseases [12, 13].

In this study, we hypothesized that IL-17A expression is elevated during *A*. *cantonensis* infection and that anti-IL-17A antibody can ameliorate the demyelination in infected animals. In this study, we report that IL-17A expressions were detected during *A*. *cantonensis* infection. Moreover, IL-17A-neutralizing antibody protects against demyelination caused by *A*. *cantonensis* infection. Our results showed that IL-17A and iNOS RNA expressions increased gradually in the process of *A*. *cantonensis* infection, and IL-17A inhibition alleviated the demyelination caused by *A*. *cantonensis.* Furthermore, we also report that IL-17A inhibition may decrease the production of iNOS, which might be the key reason for the curative effect of IL-17A-neutralizing antibody on the demyelination caused by *A*. *cantonensis.* These findings explore a role of IL-17A on the demyelination caused by *A*. *cantonensis* and provide a new potential alternative therapy for this disease.

## 2. Materials and Methods

### 2.1. Infection of Mice with *A. cantonensis* Larvae and IL-17 Antibody Injection

Mice infected with *A. cantonensis* larvae BALB/c mice (20–40 g body weight) were purchased from the Animal Center Laboratory at Sun Yat-sen University (Guangzhou, China). The Institutional Animal Care and Use Committee approved all animal procedures. Larval collection: stage III larva (L3) of *A. cantonensis* were collected from giant African snails (*Achatina fulica*) via homogenization and digestion of minced snail tissue that was placed in a pepsin-HCl solution (pH 2.0, 500 IU pepsin/gram tissue) and incubated at 37°C for 2 h. L3 in the sediment were washed with phosphate-buffered saline (PBS) and counted under an anatomical microscope then given to experimental animals by gavage with 30 L3 per animal. The animals were divided into four groups: normal control group, normal control with IL-17 antibody, *A. cantonensis* infection group, and *A. cantonensis* infection group treated with IL-17 antibody. There are at least 5 mice in each group. The mice in the normal control treated with IL-17 antibody group and in the *A. cantonensis* infection treated with IL-17 antibody group were injected with IL-17-neutralizing antibody (per 0.05 mg/kg/day; eBioscience, USA) into the abdominal cavity at 0 d, 4 d, 8 d, 12 d, 16 d, and 20 d.

### 2.2. Transmission Electron Microscopy Observation

After anesthesia, the animals were euthanatized by transcardial perfusion with 4% paraformaldehyde. Mice' optic nerves were crosscut into 15 *μ*m sections at −20°C and mounted on glass slides. Optic nerves were quickly dissected and postfixed overnight in 2.5% glutaraldehyde. Next, optic nerve fragments were postfixed in a solution containing 1% osmium tetroxide (Sigma-Aldrich), then fragments were dehydrated in acetone series and embedded in SPIN-PON resin. Resin polymerization was performed at 60°C for three days. Semithin sections (0.5 *μ*m thickness) were placed onto glass slides and stained with toluidine blue. Finally, demyelination detection was done by using a 300 kV transmission electronic microscope (FEI, USA).

### 2.3. Immunofluorescence

After fixing with 4% paraformaldehyde, brain sections were cut into 15 *μ*m sections at −20°C and mounted on glass slides. Then, sections were blocked with 3% bovine serum albumin (BSA) at room temperature for 1 h before incubation with rabbit anti-iNOS (Abcam, UK) and anti-GFAP (Sigma-Aldrich, USA) monoclonal antibody in 1% BSA at 4°C overnight. Sections were washed three times in PBS, incubated with fluorescein isothiocyanate- (TRITC-) labeled and FITC-labeled (for others) secondary antibody(Abcam, Cambridge, UK), diluted 1 : 500 in 1% BSA at 37°C for 1 h, and washed again in PBS. Then, DAPI (1 : 1000 dilution, Beyotime Biotechnology) stained the nucleus for 5 min. Specimens stained without the primary antibody were used as negative controls. Then, the slides were observed under a confocal microscope.

### 2.4. RNA Isolation and Real-Time Quantitative PCR

Total RNA was extracted from the cerebrum with TRIzol reagent according to the manufacturer's instructions (Invitrogen). For cDNA synthesis, RNA was reverse transcribed with a PrimeScript RT reagent Kit (TaKaRa). The expression of the genes encoding IL-17A, and iNOS for mice by real-time PCR with SYBR Premix Ex Taq kit (TaKaRa). Relative quantification was applied to detect the mRNA expression of the above genes. The primer sequences as follows: 5′-TCATGTGGTGGTCCAGCTTTC-3′, 3′-CTCAGACTACCTCAACCGTTCC-5′ for IL-17A mice; 5′-CTGATGTTGCCATTGTTGGTG-3′, 3′-CTTTGACGCTCGGAACTGTAG-5′ for iNOS mice; and 5′-AAGAAGGTGGTGAAGCAGG-3′; 3′-GAAGGTGGAAGAGTGGGAGT-5′ for GAPDH mice as an internal reference. Amplification of cDNA was performed on an ABI Prism 7900 HT cycler (Applied Biosystems).

### 2.5. Western Blot Analysis

The brain tissue of mice in different groups were washed twice with cold PBS and lysed in extraction buffer (20 mM HEPES, pH 7.4, 2 mM EDTA, 50 mM 5-glycerophosphate, 1 mM dithiothreitol, 1 mM Na_3_VO_4_, 1% Triton X-100, and 10% glycerol) on ice. The lysates were centrifuged at 12,000 rpm for 15 min, and supernatants were collected. Protein (20–40 *μ*g) was separated by SDS-PAGE and then transferred onto a nitrocellulose membrane (Pall Corporation, Ann Arbor, MI, USA). Transferred blots were incubated sequentially with a blocking agent (5% nonfat milk in TBS), and an anti-MBP antibody (1 : 125 dilution, Abcam, UK) and a HRP-conjugated secondary antibody (for 1 h at room temperature) were developed by the enhanced chemifluorescence detection kit on Hyperfilm (Fuji, Japan) according to the manufacturer's directions. The same blots were subsequently stripped and reblotted with internal referring antibodies *β*-actin and *β*-tubulin (Sigma-Aldrich, USA). Graphs of blots were obtained in the linear range of detection and were quantified for the level of specific induction by ImageJ System.

### 2.6. Statistical Analysis

One-way ANOVA was used to compare data of real-time PCR and graphs of blots in western blotting among different groups. Statistics was performed using IBM SPSS statistics 19 (SPSS Inc., USA). *P* < 0.05 was considered statistically significant.

## 3. Results

### 3.1. Demyelination Is of Serious Pathological Change in the Brain Tissue of Mice with *A. cantonensis* Infection

**Figure 1:**
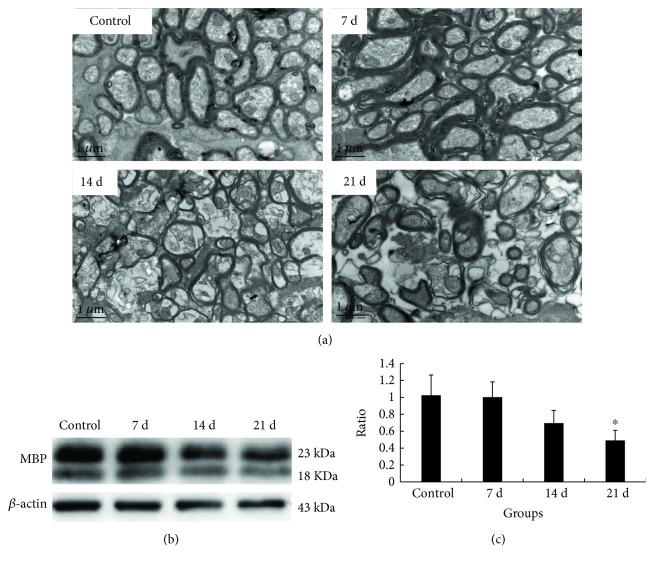
*A*. *cantonensis* infection caused demyelination in infected mice. (a) Transmission electronic image of the optic nerve at 0 d, 7 d, 14 d, and 21 d after *A*. *cantonensis* infection. Prominent demyelination (the black arrows point) can be observed in 14 d and 21 d. Scale bar = 1 *μ*m. (b) The MBP protein expression of the brain via western blotting at 0, 7, 14, and 21 days after *A*. *cantonensis* infection. (c) The relative ration of MBP protein expression (*β*-actin was as the internal reference). These expressions decreased as time extended and had obvious difference with the control in 21 d of infection. Numerical results are presented as mean ± SEM. *n* = 3 animals per group, at least three fields were analyzed per section in at least two sections not next to each other per animal. ∗ represents statistically significant values when compared with the normal control (*P* < 0.05).

*A. cantonensis* invaded the central nerve system and caused demyelination. The resulting images of the transmission electronic microscope of the optic nerves showed that demyelination was obvious at 14 d and became serious on 21 d of infection of *A. cantonensis* (Figure 1(a)). Moreover, the MBP (myelin basic protein) expression also decreased gradually during *A. cantonensis* infection (Figures 1(b) and 1(c)). The above results proved that *A*. *cantonensis* can cause demyelination in the brain.

### 3.2. Increase of IL-17A and iNOS Is Significant in the Brain of the Infected Mice

**Figure 2:**
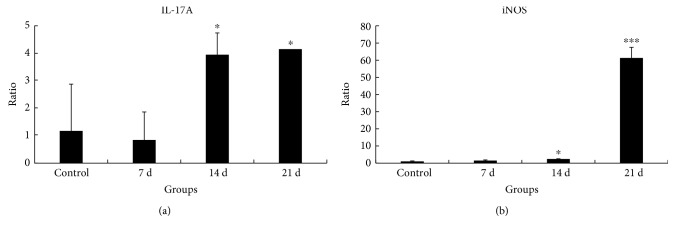
*A*. *cantonensis* induced the elevation of IL-17A and iNOS. The RNA expression of IL-17A and iNOS at 0 d, 7 d, 14 d, and 21 d after *A*. *cantonensis* infection (GAPDH was the internal reference for real-time PCR). (a) Expression of IL-17A increased gradually. *n* = 3 animals per group. (b) RNA expression of iNOS, which also elevated with infection time obviously. ∗ and ∗∗∗ represent statistically significant values when compared with normal control (*P* < 0.05 and *P* < 0.001, resp.).

We further explored whether the expression of IL-17A is altered in the process during *A*. *cantonensis* infection. We found that IL-17A RNA expression increased with the extension of the infection (Figure 2(a)). At the same time, the RNA expression of iNOS also increased gradually and peaked at 21 d of infection (Figure 2(b)). These findings suggest that IL-17A and iNOS may correlate with demyelination caused by *A*. *cantonensis*.

### 3.3. IL-17A Inhibition Can Lighten the Demyelination in the Brain of the Infected Mice

**Figure 3:**
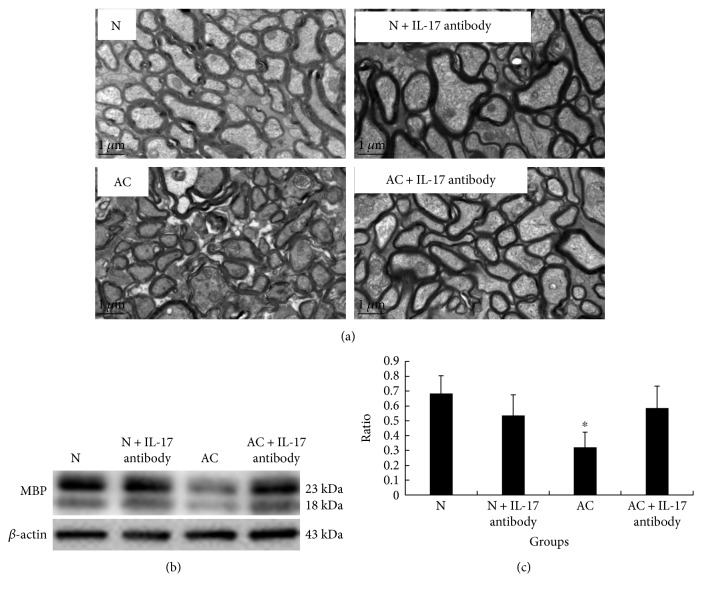
IL-17A inhibition can ameliorate the demyelination caused by *A*. *cantonensis.* (a) TEM picture of the optic nerve in the normal group, 21 d *A*. *cantonensis* infection group, normal mice with IL-17A antibody group, and 21 d *A*. *cantonensis* infection with IL-17A antibody group. IL-17A inhibition can restore the demyelination caused by *A*. *cantonensis* to the normal level. Scale bar = 1 *μ*m. (b) The MBP protein expression of the brain via western blotting in the normal group, 21 d *A*. *cantonensis* infection group, normal mice with IL-17A antibody group, and 21 d *A*. *cantonensis* infection with IL-17A antibody group. (c) The semiquantification of MBP protein expression via western blotting in different groups (*β*-actin as the internal reference). IL-17A inhibition had no effect on MBP expression of the brain in the normal group but increased MBP expression in the 21 d *A*. *cantonensis* infection group. *n* = 3 animals per group, at least three fields were analyzed per section in at least two nonadjacent sections per animal. ∗ represents statistically significant values when compared with normal control (*P* < 0.05).

TEM and MBP protein expressions were applied to detect the alteration of demyelination. When *A*. *cantonensis* infected the mice for 21 d, TEM showed obvious demyelination. Moreover, MBP expression also decreased distinctly. After IL-17A antibody was injected in experimental mice, the results showed that IL-17A inhibition can restore the demyelination caused by *A*. *cantonensis* to normal levels (Figure 3(a)). In addition, levels of MBP and expression were elevated after IL-17A inhibition of *A. cantonensis* in infected mice but had no effect on normal mice (Figures 3(b) and 3(c)). These results displayed that IL-17A may be the key element for demyelination caused by *A. cantonensis*.

### 3.4. IL-17A Inhibition Can Cause the Downregulation of iNOS in Astrocytes in the Brain of the Infected Mice

**Figure 4:**
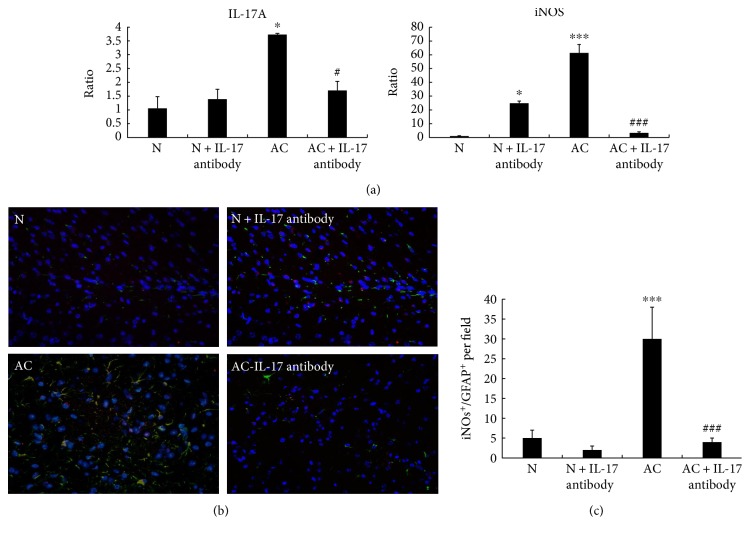
IL-17A inhibition causes the downregulation of iNOS in astrocytes after *A. cantonensis* infection. (a) The RNA expression of IL-17A and iNOS in the normal group, the 21 d *A*. *cantonensis* infection group, the normal mice with IL-17A antibody group, and the 21 d *A. cantonensis* infection with IL-17A antibody group. After IL-17A antibody was injected, the RNA expression of IL-17A was decreased in the *A*. *cantonensis*-infected group on day 21 but it had no effect on the normal group. IL-17A antibody increased the RNA expression of iNOS in the normal group, but levels were decreased in the 21 d *A*. *cantonensis* infection group. GAPDH was the internal reference for real-time PCR. (b) Brain sections stained with iNOS (red) and GFAP (green). Yellow color represented the double staining of iNOS and GFAP. (c) Percentage of iNOS^+^/GFAP^+^ cells per field of the brain section. ^∗^*P* < 0.05, ^∗∗∗^*P* < 0.001, compared with the normal group; ^#^*P* < 0.05, ^###^*P* < 0.001, compared with the 21 d *A*. *cantonensis* infection group. *n* = 3 mice.

From above results, we found that IL-17A inhibition can alleviate demyelination caused by *A*. *cantonensis*, but the mechanism was still unknown. To resolve this question, real-time PCR and immunofluorescence were applied to examine the expression of IL-17A and iNOS. Our results showed when *A. cantonensis* infected the mice for 21 d, IL-17A and iNOS elevated obviously. IL-17A-neutralizing antibody can decrease the expression of IL-17A, which proved that the inhibition of IL-17A in *A*. *cantonensis*-infected mice was effective, but it cannot influence the IL-17A expression of normal animals. More importantly, iNOS expression was also decreased obviously during IL-17A inhibition. Moreover, we found that when IL-17A-neutralizing antibody was applied, iNOS expression in normal mice was elevated (Figure 4(a)). The results of immunofluorescence also proved that when *A*. *cantonensis* infected the mice for 21 d, the double labeling of iNOS and GFAP (astrocytes marker) both increased, whereas IL-17A inhibition can decrease the protein expression of iNOS in astrocytes (Figures 4(b) and 4(c)). The above results showed that IL-17A inhibition may alleviate demyelination caused by *A. cantonensis* through decreasing the iNOS expression in astrocytes.

## 4. Discussion

*A. cantonensis* is an important cause of food-borne diseases in humans. *A. cantonensis* infection is endemic in the Pacific Islands, Southeast Asia, China, and Hawaii. Sporadic infections are reported in Southern United States and Florida [14]. Outbreaks of eosinophilic meningitis have been reported due to the consumption of raw snails harboring L3 or associated with the consumption of contaminated raw vegetable juice in Taiwan [15, 16]. Rat is a known definitive host which can tolerate the worms. In large numbers (240), *A. cantonensis* cause significant cardiovascular and neurological impairment in rats [17].

In this study, we detected IL-17A expression in the brain tissue of the infected mice and applied IL-17A-neutralizing antibody to treat the infected mice to investigate the process of brain demyelination. Our findings demonstrated that *A. cantonensis* infection promotes the IL-17A expression in the brain as well as IL-17A inhibition to alleviate the demyelination caused by *A*. *cantonensis* in mice. Moreover, IL-17A inhibition may decrease the production of iNOS, which might be the reason for the curative effect for IL-17A-neutralizing antibody on demyelination caused by *A. cantonensis*. The evidence was as follows.

Firstly, TEM detection of the optic nerve and western blotting of MBP proved that *A*. *cantonensis* infection actually caused demyelination. Moreover, the RNA expression of IL-17A and iNOS increased gradually following with the prolonged *A. cantonensis* infection. IL-17 is a mediator of communication between immune cells and tissues. IL-17 is the founding member of a family of 6 cytokines, IL-17A–F, and IL-17A is the most investigated one. IL-17A has recently emerged as an attractive target, especially for the treatment of T cell-mediated autoimmune diseases [18, 19]. Previous study showed that IL-17A expression alone was able to activate glial cells and enhance neuroinflammatory responses, thus showing that CNS cells express a functional IL-17RA/C receptor complex [6]. In our study, IL-17A expression elevated with demyelination during *A*. *cantonensis* infection. Similarly, we observed elevation of iNOS associated with demyelination in experimental animals. These findings indicate that IL-17A along with iNOS may take part in the demyelination caused by *A*. *cantonensis.*

Next, the IL-17A-neutralizing antibody was administered in the infected mice to explore the function of IL-17A on demyelination caused by *A*. *cantonensis*. Our results proved that IL-17A inhibition can alleviate demyelination obviously observed via TEM of the optic nerve and western blotting of MBP after *A*. *cantonensis* infection. In the previous study, there were strong indications pointing to a role for IL-17 in the pathogenesis of MS and EAE, major causes of demyelination diseases [20, 21]. As a result, we applied IL-17A-neutralizing antibody to treat the demyelination caused by *A*. *cantonensis* and the effect proved our presumption.

Finally, we explored the mechanism by which IL-17A-neutralizing antibody alleviated demyelination caused by *A*. *cantonensis*. Our results showed that IL-17A-neutralizing antibody inhibited the RNA expression of IL-17A and also decreased the production of iNOS induced by *A*. *cantonensis* infection. Moreover, IL-17A-neutralizing antibody also inhibited the protein expression of iNOS in astrocytes. Astrocytes probably represent the best-studied CNS resident cell type in the context of MS and EAE. Both human and mouse astrocytes express IL-17RA, thereby allowing for IL-17A ligation and consequently, the production of cytokines and chemokines, including IL-6, TNF*α*, and CCL2 [6, 7]. iNOS is involved in various physiological regulations and plays important roles in some CNS disease, such as brain ischemia, brain infections, and neurodegenerative diseases [22, 23]. As a result, we speculated that IL-17A-neutralizing antibody alleviated demyelination via inhibiting iNOS production in astrocytes. Previous study showed that exogenous IL-17A significantly induced iNOS expression and hence cardiomyocyte apoptosis [24]. Our study was partly in accordance with these findings. To the best of our knowledge, there has been no previous report that IL-17A inhibition would decrease the production of iNOS in demyelination caused by *A*. *cantonensis.* In this line, we will further investigate iNOS using knockout mice in future studies.

In conclusion, IL-17A may play important roles in demyelination caused by *A*. *cantonensis* and the present study suggested that IL-17A-neutralizing antibody may be an effective method to treat the demyelination caused by *A*. *cantonensis*. Furthermore, iNOS inhibition is the possible mechanism for the therapeutic effect. As a result, our study provides a new potential alternative therapy for demyelination caused by *A. cantonensis*.
