# Corrigendum to “Inhibiting Interleukin 17 Can Ameliorate the Demyelination Caused by *A. cantonensis* via iNOS Inhibition”

**DOI:** 10.1155/2018/9098287

**Published:** 2018-05-02

**Authors:** Ying Feng, Cunjing Zheng, Feng Feng, Shuo Wan, Xin Zeng, Fukang Xie, Zhongdao Wu

**Affiliations:** ^1^Medical School of South China University of Technology, Guangzhou, Guangdong 510006, China; ^2^Histology and Embryology Department of Zhongshan School of Medicine, Sun Yat-sen University, Guangzhou 510080, China; ^3^The Department of Pharmacology and Toxicology, School of Pharmaceutical Sciences, Sun Yat-sen University, Guangzhou 510080, China; ^4^Parasitology Department of Zhongshan School of Medicine, Sun Yat-sen University, Guangzhou 510080, China

In the article titled “Inhibiting Interleukin 17 Can Ameliorate the Demyelination Caused by *A. cantonensis* via iNOS Inhibition” [1], the names of the first, second, fourth, sixth, and seventh authors were reversed. The revised authors' list is shown above.
